# Isolation and characterization of *Candida tropicalis* B: a promising yeast strain for biodegradation of petroleum oil in marine environments

**DOI:** 10.1186/s12934-023-02292-y

**Published:** 2024-01-13

**Authors:** Ghada E. Hegazy, Nadia A. Soliman, Soha Farag, Ehab R. El-Helow, Hoda Y. Yusef, Yasser R. Abdel-Fattah

**Affiliations:** 1 National Institute of Oceanography & Fisheries, NIOF-Egypt, Qaitbay Sq, El-Anfoushy, Alexandria, 11865 Egypt; 2 Bioprocess Development Department, Genetic Engineering & Biotechnology Research Institute (GEBRI), City of Scientific Research & Technological Applications (SRTA-City), New Borg Elarab City, Alexandria, Egypt; 3 Environmental Biotechnology Department, Genetic Engineering & Biotechnology Research Institute (GEBRI), City of Scientific Research &Technological Applications (SRTA-City), New Borg Elarab City, Alexandria, Egypt; 4 Botany & Microbiology Department, Faculty of Science, Alexandria University, Alexandria, Egypt

**Keywords:** Oil biodegradation, *Candida tropicalis*, Statistical experimental design, Immobilization

## Abstract

The increasing interest in environmental protection laws has compelled companies to regulate the disposal of waste organic materials. Despite efforts to explore alternative energy sources, the world remains heavily dependent on crude petroleum oil and its derivatives. The expansion of the petroleum industry has significant implications for human and environmental well-being. Bioremediation, employing living microorganisms, presents a promising approach to mitigate the harmful effects of organic hydrocarbons derived from petroleum. This study aimed to isolate and purify local yeast strains from oil-contaminated marine water samples capable of aerobically degrading crude petroleum oils and utilizing them as sole carbon and energy sources. One yeast strain (isolate B) identified as *Candida tropicalis* demonstrated high potential for biodegrading petroleum oil in seawater. Physiological characterization revealed the strain’s ability to thrive across a wide pH range (4–11) with optimal growth at pH 4, as well as tolerate salt concentrations ranging from 1 to 12%. The presence of glucose and yeast extract in the growth medium significantly enhanced the strain's biomass formation and biodegradation capacity. Scanning electron microscopy indicated that the yeast cell diameter varied based on the medium composition, further emphasizing the importance of organic nitrogenous sources for initial growth. Furthermore, the yeast strain exhibited remarkable capabilities in degrading various aliphatic and aromatic hydrocarbons, with a notable preference for naphthalene and phenol at 500 and 1000 mg/l, naphthalene removal reached 97.4% and 98.6%, and phenol removal reached 79.48% and 52.79%, respectively. Optimization experiments using multi-factorial sequential designs highlighted the influential role of oil concentration on the bioremediation efficiency of *Candida tropicalis* strain B. Moreover, immobilized yeast cells on thin wood chips demonstrated enhanced crude oil degradation compared to thick wood chips, likely due to increased surface area for cell attachment. These findings contribute to our understanding of the potential of *Candida tropicalis* for petroleum oil bioremediation in marine environments, paving the way for sustainable approaches to address oil pollution.

## Introduction

Marine pollution is the contamination of oceans and seas with harmful substances, leading to detrimental effects on marine life and ecosystems. It is caused by industrial activities, improper waste disposal, oil spills, agricultural runoff, and plastic waste. This pollution poses significant threats to biodiversity, human health, and the overall health of the planet. It is crucial to address this issue through stricter regulations, sustainable waste management, promoting eco-friendly alternatives, raising awareness, and fostering international cooperation. By taking collective action, we can protect our oceans and ensure a sustainable future for marine ecosystems. Water pollution with petroleum oil and its derivatives is one of a widespread and a serious ecological hazard problem. Using the petroleum oil and its organic derivatives in addition to their transportation through the seas, has made them major pollutants and contaminants in the marine environment. The oil spill impact can be understood that one crude oil barrel can make water undrinkable barrels [1]. The effect of petroleum pollution on marine organisms are multifaceted. Oil spills coat marine surfaces, impairing the function of feathers and fur of marine birds and mammals, leading to reduced insulation and buoyancy [2] Additionally, oil can penetrate the gills and respiratory distress and impairing their ability to extract oxygen from water. The toxic components of petroleum such as total petroleum hydrocarbons (TPH) and polycyclic aromatic pollutants (PAHs) which affect on the public living organisms’ health, especially in oil-rich and developing countries such as Egypt, which persist in the environment and can accumulate in the tissues of marine organisms, leading to bioaccumulation and biomagnification within the food web. Furthermore, the interplay between marine pollution and petroleum pollution extends beyond immediate ecological consequences. Climate change exacerbates the impacts of oil spills, as rising sea temperatures and changing ocean currents influence the spread and behavior of spilled oil. Additionally, the combination of petroleum pollution and other pollutants, such as plastics and chemical contaminants, creates synergistic effects that intensify the overall ecological harm to marine ecosystems [3–5]. TPHs represent a large family of organic and chemical compounds that were derive from crude petroleum oil. Various TPH compounds can be separated from the crude mixture according to their chemical properties such as evaporation and dissolving in water. TPHs include hydrocarbons with high molecular weight, while PAHs include toxic oil hydrocarbons. PAHs represent carcinogenic hydrocarbons species. PAHs comes into the environment by two ways natural processes and anthropogenic sources. Anthropogenic sources include both pyrogenic and petrogenic ones. Petrogenic PAHs such as spills of petroleum oil-derived products and dispersion of organic compound in anoxic marine environment. Pyrogenic PAHs originates from incomplete fossil fuels combustion, municipal and biomass wastes [6]. The contamination of water by hydrocarbons causes serious damage to the life in the environment, for example the pollutants accumulation in the plant and animals tissues can lead to mutations or complete death of the living organism [7]. Abu-Qir gulf, Alexandria, Egypt is subjected to pollution and contamination by various and different organic contaminants, including organic hydrocarbons discharges from larges boat tank spills. This pollution has a serious and hazard impact on all economic, heath, and environmental aspects. Biodegradation is an alternative method that has can be used to decrease organic hydrocarbons pollutants in the marine environment by using microorganisms as degradative machinery. This method is accepted worldwide as effective and eco-friendly useful treatment that results to complete mineralization of the organic hydrocarbons pollutants at a low cost [8]. Microorganisms play a crucial role in the biodegradation of oil spills, serving as nature's own cleanup crew. Certain microorganisms, such as bacteria, fungi and yeasts have evolved the ability to break down and metabolize hydrocarbons found in crude oil and petroleum-based products. These oil-degrading microorganisms possess specialized enzymes that can efficiently break down complex hydrocarbon molecules into simpler compounds, which can then be utilized as a source of energy and carbon for their growth. They thrive in oil-contaminated environments, where they multiply rapidly and form biofilms that enhance their degradation capabilities. Through their metabolic activities, these microorganisms help mitigate the environmental impact of oil spills by accelerating the natural process of biodegradation and reducing the persistence of oil pollutants. The use of these microorganisms in bioremediation strategies has proven to be an effective and environmentally friendly approach for restoring ecosystems affected by oil spills fungi [9, 10]. Walker et al., reported that using yeasts as degraders of crude petroleum oil and its organic hydrocarbon derivatives is preferred than other microorganisms [11]. In addition, Obuekwe et al., reported that yeasts can utilize n-alkanes as a sole energy and carbon source [12, 13]. Many yeast species, such as *Geotrichum* sp., *Candida lipolytica*, *Trichosporon mucoides* and *Yorrow lipolytica,* isolated and purified from polluted water, were reported to their ability to degrade the petroleum oil pollutants [14, 15]. Also, Benmessaoud et al., studied the diversity and biotypology of the yeasts in this region, which were used as a good indicator for the disturbance of the ecosystem by crude oil pollution [16]. However, this needs to explore more biodegradation process of different organic hydrocarbons. In this sense, to deepen and enrich the biodegradation acknowledge of marine yeasts, especially the regions contaminated by organic hydrocarbons pollutants and crude oil, a study was carried on Abu-Qir gulf the most polluted area in the Mediterranean Sea in Egypt. The objectives of this study depends on isolation of aerobic hydrocarbons degrading marine yeasts from Abu-Qir (hydrocarbons polluted site), also identification the isolates using molecular technique on the species level and finally to evaluate the biodegradation process by using statistical experimental design and immobilization process.

## Material and methods

### Sample collection and isolation of marine oil degrading microorganisms

Samples (Seawater and slurry sediments) were collected from Abu Qirgulf, which is highly polluted with heavy oil resulted from fisher boats. Samples were enriched in nutrient broth medium dissolved in sea water (with initial pH 6.5) and incubated at 30 °C for three days. The enriched cultures dissolved in sea water, synthetic sea water agar (SSWA) and natural sea water agar (NSWA) supplemented with 1% glucose. Yeast colonies were selected from plates and purified by re-plating on agar plates. Colonies were reselected to check their shape. Only cultures with a single shape were selected. All media used were prepared and sterilized by autoclaving at 121 °C for 20 min. The following media were used throughout the work. MP medium: Malt extract 20.0 g; peptone 5.0 g; and distilled H_2_O was added to 1 L.GPY medium: Glucose 20.0 g; peptone 10.0 g; yeast extract 5.0 g; and distilled H_2_O were added to 1 L. MMGY medium: Malt extract 6.0 g; maltose 1.8 g; glucose 6.0 g; yeast extract 1.2 g; and distilled H2O was added to 1 L. Natural Sea Water Agar: Filtered sea water 1000 ml; agar 20 g. M1: Sea water + 0.5% oil. M2: Sea water + 1.0% oil. M3: Sea water + 0.5% oil + 0.5% glucose. M4: Sea water + 0.5% oil + 0.5% yeast extract. M5: Sea water + 0.5% oil + 0.5% glucose + 0.5% yeast extract. M6: Distilled water + 2% malt extract + 0.5% peptone [17].

### Molecular identification of the isolated strain

The 18 s rRNA gene was amplified from genomic DNA as described by Cai et al*.,* (1996). A 1200 base-pair fragment of the 18S rRNA gene was PCR amplified by a using the following primer sequences:

**F (149):** 5′GGAAGGG (G/A) TGTATTTATTAG 3′

**R (1709):** 5′TCCTCTAAATGACCAAGTTTG 3′

The PCR mixture contained 5.0 μl of 10 × PCR buffer, 5.0 μl of 200 μM dNTPs, 5.0 μl template DNA, 3.0 μl of 10 pM of forward and reverse primers, 1.5 μl of 50 mM MgCl_2_ and 0.5 μl Taq polymerase (5 U/μl). Finally the total volume was completed up to 50 μl with ddH_2_O. The amplification program was set for 30 cycles of denaturation at 94 °C for 1 min, annealing at 55 °C for 1 min and extension at 72 °C for 2 min. One fifth of the reaction was analyzed on 1.5% agarose gel and visualized after ethidium bromide staining on a UV transilluminator. Afterwards the products were submitted in the central lab at City of Scientific Research & Technological Applications (SRTA-city), New Borg El Arab city, Alexandria, Egypt for DNA sequencing followed by phylogenetic analysis. Sequence similarities and phylogenetic analysis: The Blast program (www.ncbi.nlm.nih.gov/blast) was used to assess the DNA similarities. Multiple sequence alignment and creation of phylogenetic tree was performed using BioEdit and Tree View software [18].

### Analysis of residual hydrocarbons

#### High pressure liquid chromatography (HPLC) of aromatic hydrocarbons

HPLC was used for the quantification of biodegradation residuals of hydrocarbons which are extracted from the culture medium by solvent extraction method and carried out using Beckman system Gold 126 Solvent Module, 168 Detector (Dioale array) and outosampler (507e). Both naphthalene and naphthylamine were extracted from cultures by hexane while pentadecane, pentane, hexane, heptane, hexadecane, phenol and phenanthrene were extracted by dichloromethane. The extracted hydrocarbons were injected with standards to the Column 250 X 4.6 mm, hyper clone 5 µL ODSC18 at automatic injection 20 µL/wave length 254 nm, mobile phase (75% acetonitryl, 25% water), and the oven temperature was maintained at 150 °C for naphthalene, naphthylamine and pentadecane and 200 ºC for phenanthrene. The split ratio was 50:1 and split injection was 1.0 µl [19, 20].

#### Investigation of the degradation potency of the selected strain on different naphthalene and phenol concentrations.

Volumes of 100 ml sea water medium containing different concentrations of naphthalene and phenol ranged between (500 to 3000 mg/L) were inoculated by 2% of yeast pre-culture and incubated at 30 °C with a shaking speed of 200 rpm for 3 days. The degradation efficiency was monitored by measuring OD and residual naphthalene and phenol concentrations [19].

### Optimization of petroleum oil consumption by *C. tropicalis* strain B

#### The Plackett–Burman design

The used independent variables were screened according to the Plackett and Burman design (PBD) [21]. All trials were achieved in triplicate and the average of oil consumption yield were treated as responses. The main effect of each variable was calculated as Plackett–Burman experimental design is based on the first order model:

$$ {\text{Y}}\, = \,\beta 0\, + \,\Sigma \, \beta {\text{ixi}}. $$

where Y is the response (oil consumption), β0 is the model intercept and βi is the variables. Statistical software, such as MICROSOFT EXCEL can be used to perform a statistical data analysis. Therefore, two times the factor estimate represents the changes in titer over the range (− 1 to + 1). The change in the response over the entire range is called the main effect of a given factor. either positive or negative, indicates that a factor has a large impact on titer; while an estimate close to zero means that a factor has a little or no effect. The *P*-value is the probability that the magnitude of a parameter estimate is due to random process variability. At low* P*-value indicates a “real” or significant effect.

#### Box-Behnken design (BBD) and data analysis

An experimental design was applied to find out the optimum level of effective variables [21]. The most significant variables were selected for further optimization experiment of their optimal level with respect to the oil consumption as response. This was performed through a quadratic model and determining true values of model coefficient in 13 trial design matrix for strain B., the most significant variables were selected for further determination of their optimal level. For this reason, Box-Behnken design, which is a response surface methodology, was applied. The factors of highest confidence levels as showed through Plackett–Burman experimental design, D-glucose (X_1_); oil (X_2_) and pH (X_3_) were prescribed into three levels coded − 1, 0, and + 1. The three variables (each at its three levels) were tested in 13 different combinations.

For predicting the optimal point, a second order polynomial function was fitted to correlate relationship between independent variables and oil consumption for strain B.

For the three factors, the following equation was used:

$$ {\text{Y}}_{{\text{B}}} \, = \,\beta_{0} + \beta_{{1}} {\text{X}}_{{1}} \, + \,\beta_{{2}} {\text{X}}_{{2}} \, + \,\beta_{{3}} {\text{X}}_{{3}} \, + \,\beta_{{{12}}} {\text{X}}_{{1}} {\text{X}}_{{2}} \, + \,\beta_{{{13}}} {\text{X}}_{{1}} {\text{X}}_{{3}} \, + \,\beta_{{{23}}} {\text{X}}_{{2}} {\text{X}}_{{3}} \, + \,\beta_{{{11}}} {\text{X}}_{{1}}^{{2}} \, + \,\beta_{{{22}}} {\text{X}}_{{2}}^{{2}} \, + \,\beta_{{{33}}} {\text{X}}_{{3}}^{{2}} . $$

Where, Y is the predicted response, β_0_ model constant; X_1_,X_2_,and X_3_ independent variables; β_1_,β_2_,and β_3_ are linear coefficients; β_12_,β_13_, and,β_23_, are cross product coefficients and β_11_,β_22_,and β_33_ are the quadratic coefficients. Microsoft Excel 97 was used for the regression analysis of the experimental data obtained. The quality of fitting of the polynomial model equation was expressed by the coefficient of determination R^2^.The data regarding oil consumption by the experimental strain were subjected to multiple regressions using Microsoft Excel to estimate t-values and *P*-values. The significance level (*P*-value) was determined using the Students t-test. The t-test for any individual effect allows an evaluation of the probability that the observed result obtained by chance. Confidence level is an expression of the *P*-value in percent. The optimal values for oil consumption were estimated using the solver function of Microsoft Excel tools [22].

#### Immobilization process

Thin wood and thick wood chips were used as carriers for the immobilization of the cells under different culturing conditions. Overnight cultures of strain B were incubated independently together with the carrier thin and thick wood chips individually. Different incubation times (1 h, 2 h, 3 h and 5 h) were tested. After cell adsorption under tested conditions, the remainders were removed by decantation. The immobilized cells were incubated under shaking conditions of 200 rpm at 30 °C for three days using natural sea water as basal medium and 1% crude oil. The efficiency of oil degradation by immobilized yeast cells under tested conditions was measured. To investigate the effect of using different weights of thin wood chips (0.5, 0.75, 1, 1.25, 1.5, 1.75 g/44cm^2^) for cell immobilization at fixed time (3 h) on oil degradation, the process was completed by adding of sterilized basal natural seawater as medium then crude oil (1%) to the immobilized cell. This was followed by incubating the tested strains at 30 °C under shaking for three days. Afterwards, the % of oil consumed was measured gravimetrically [23].

## Results

### Sampling site and isolation of yeast

The yeast isolate used in the present study was isolated from a motor oil polluted area of Abou-Qir gulf, Alexandria, Egypt, different morphotypes yeasts were selected and streaked out on NSWA supplemented with 1% glucose for purification.

### Preliminary test for degradation ability

Screening of the ability of oil degradation using the selected purified yeast colonies was performed by culturing them on NSWA plates containing petroleum oil with concentration (0.5%) and incubated at 30 °C for 7 days. Thereafter, a growing and purified colony that appeared relatively healthy was selected to continue this work and named isolate B.

### Phenotypic and molecular characterization of isolate B

**Fig. 1:**
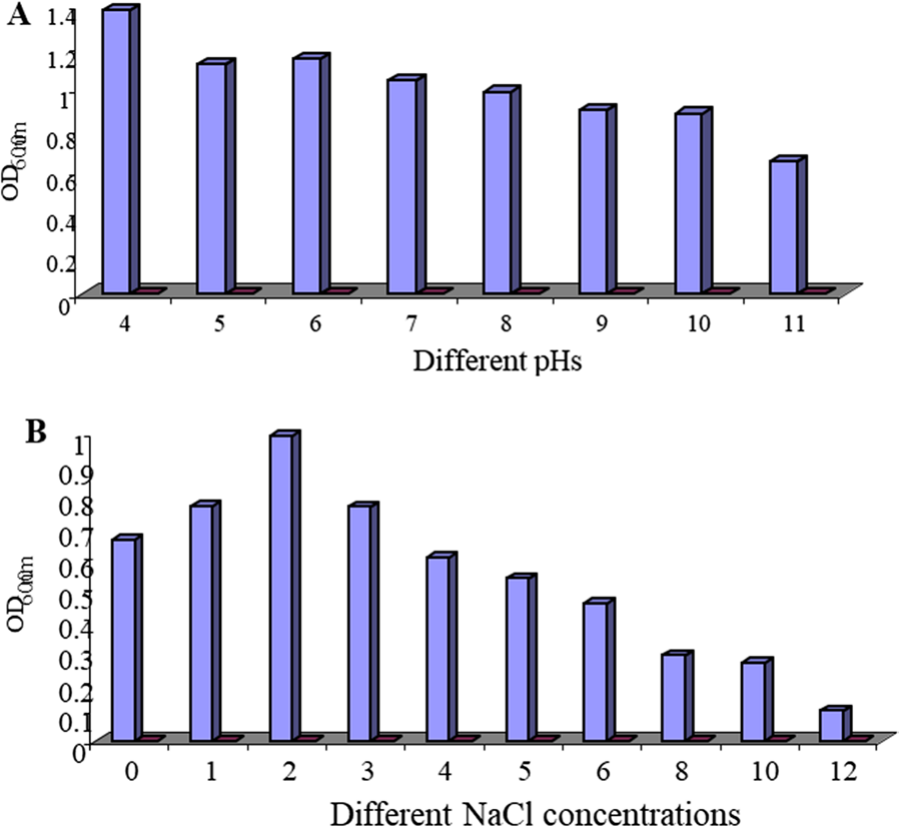
**A** Growth of isolate B at different pHs. **B** Growth of the isolate B at different NaCl concentrations

**Fig. 2:**
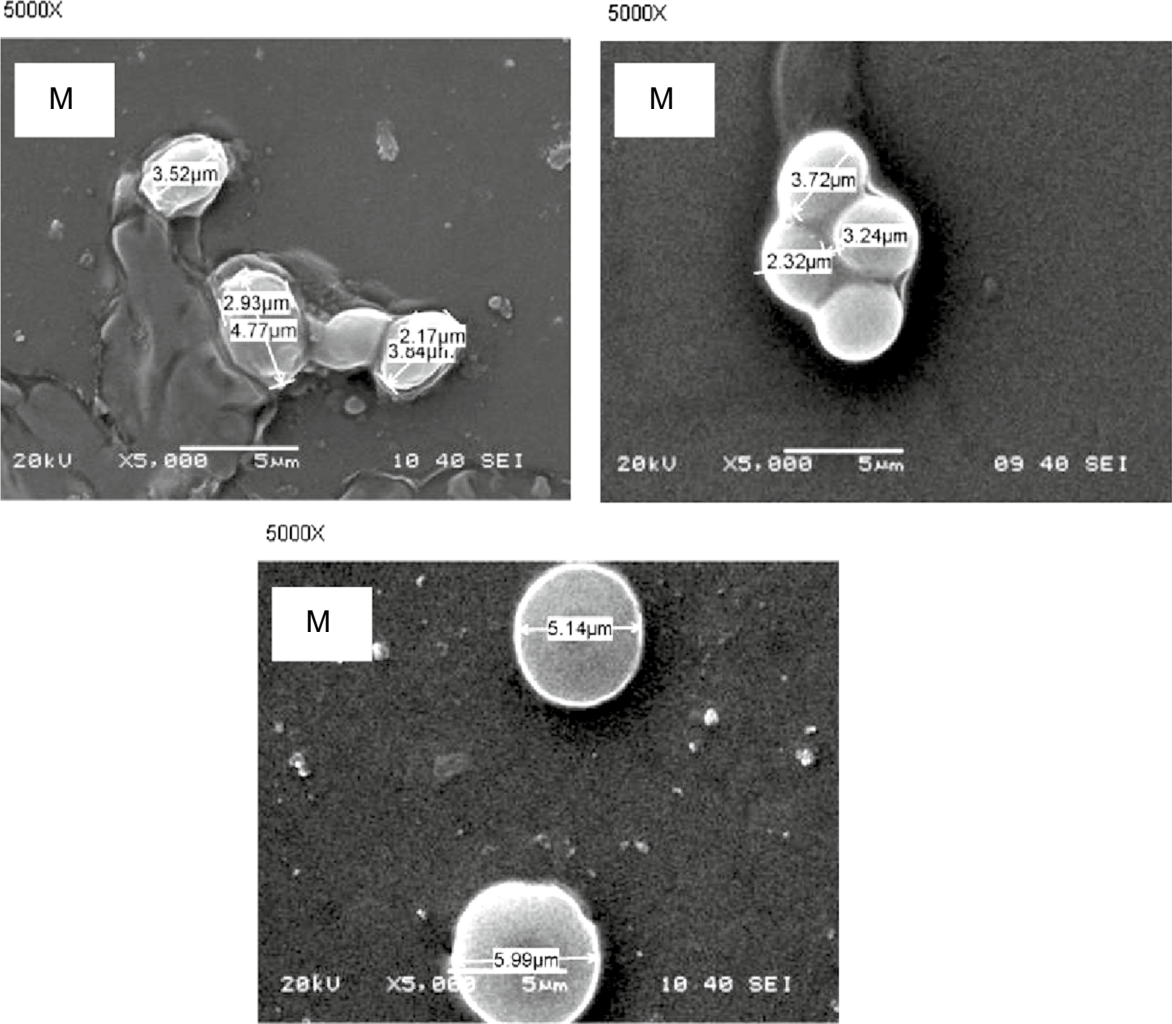
Morphology of isolate B cells grown on different media as shown by scanning electron microscopy (SEM). (M2, M5 and M6 are described in materials and methods)

For testing the growth of the selected isolate B at different variables as different temperatures, pHs values and different NaCl concentrations the yeast culture grew using nutrient broth dissolved in sea water without addition any other supplements and incubated for 3 days. two different temperatures (30 °C and 37 °C) were tested and the results obtained indicated that 30 °C was clearly better for the growth of isolate B. Also the effect of different pHs values was examined on the growth of the isolate B with different pHs values ranged from 4 to 11 pH. As shown in Fig. 1A, the selected isolate B was able to grow in all examined pHs values. Maximum growth was observed at pH 4.0, whereas the growth was progressively decreased with increasing the pH. These results indicated that the isolate B can grow at a wide range of pHs (4–11). NaCl concentrations ranging from 1 to 12% were tested at constant optimum temperature and pH. The results obtained **(**Fig. 1B**)** indicated that the best concentration for growth of the selected strain B was 2% and the growth decreased by increasing the concentration. Cellular morphology of the experimental yeast isolate grown on different media (M2, M5, and M6; and incubated for 3 days at 30 °C described in material and method) was investigated by scanning electron microscopy. Cellular dimensions were significantly changed as affected by medium composition. As shown in Fig. 2, cell diameter was maximum in the complex medium M6 (3–4 µm) and decreased was significantly smaller in M2 (2.17–3.5 µm). Intermediate sizes were observed in case of M5. The 18S rRNA of the yeast isolate B was PCR amplified from its obtained genomic DNA, an amplified band of approximately 1200 bp was obtained. The edited amplified part sequence of the gene showed a 100% identity to *Candida tropicalis* based on the alignment of the multiple sequence that was involved in the obtained sequence and close relatives. The 18S rRNA sequence of the yeast isolate B was submitted to gene bank with the accession number AY497767.

### Screening for utilization of hydrocarbons by *C. tropicalis*

**Fig. 3:**
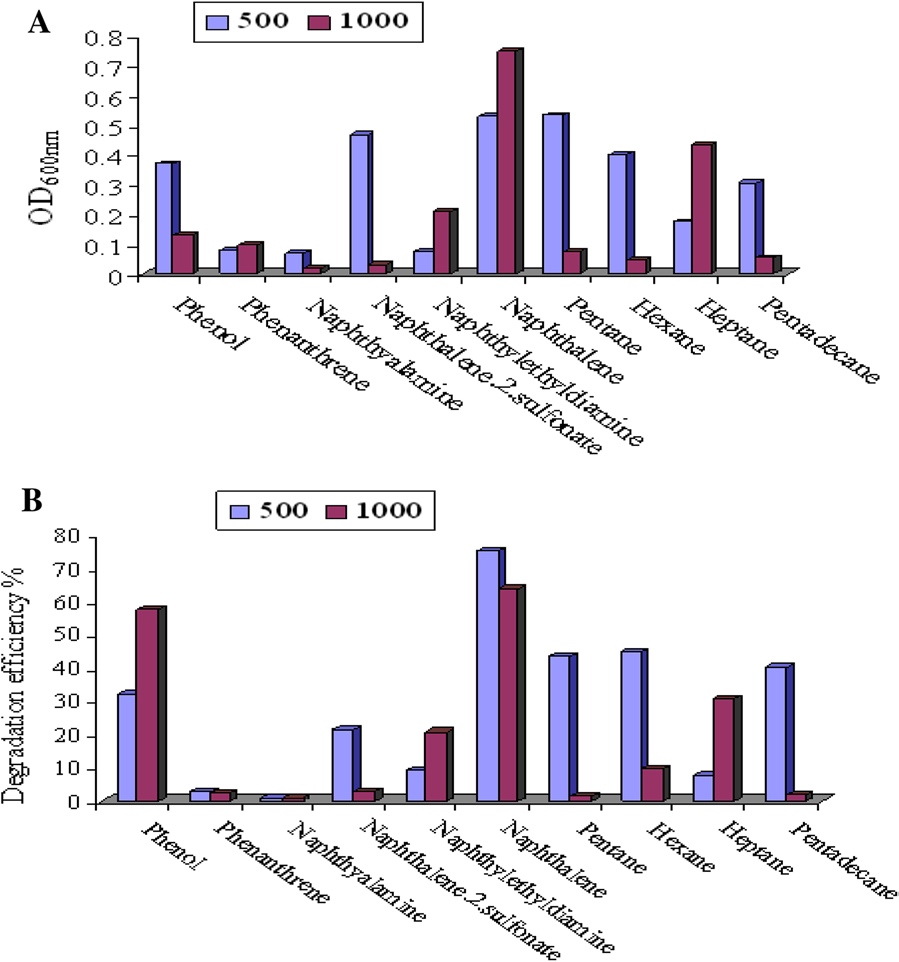
**A** Growth of *C. tropicalis* on different hydrocarbons; each was examined at the concentrations 500 and 1000 mg/l, **B** The degradation effect of *C. tropicalis* on different hydrocarbons; each was examined at the concentrations 500 and 1000 mg/l

The ability of *C. tropicalis* strain B to utilize 11 different hydrocarbons, each as a sole carbon and energy source, was examined. These included the aliphatic compounds pentane, hexane, heptane’s, pentadecane, and hexadecane and the aromatic compounds which included phenol, phenantherene, naphthyalamine, naphthalene-2-sulfonate, naphthylethyldiamine, and naphthalene. Each compound was examined at two different concentrations, specifically, 500 mg/l and 1000 mg/l in submerged natural sea water (NAW) cultures with an incubation period of 3 days. As demonstrated graphically in Fig. 3A, B, clear potential differences of growth and degradation were noticed. The results suggest that except in the cases of phenantherene, naphthylethyldiamine, naphthalene and heptane, the examined high concentration (1000 mg/l) partially inhibited the growth of the isolate. The experimental strain showed also a preference to degrade short aliphatic chains. A relatively high tendency for the utilization of naphthalene and phenol by the experimental strain was observed. At 500 and 1000 mg/l, naphthalene removal reached 97.4% and 98.6%, and phenol removal reached 79.48% and 52.79%, respectively. Considerable potencies in the degradation of pentane, hexane and pentadecane have been observed when each was introduced at a concentration of 500 mg/l. On the other hand, a slight degradation potential was recorded in the cases of naphthylamine and phenantherene.

### Degradation of different naphthalene concentrations by *C. tropicalis*

**Fig. 4:**
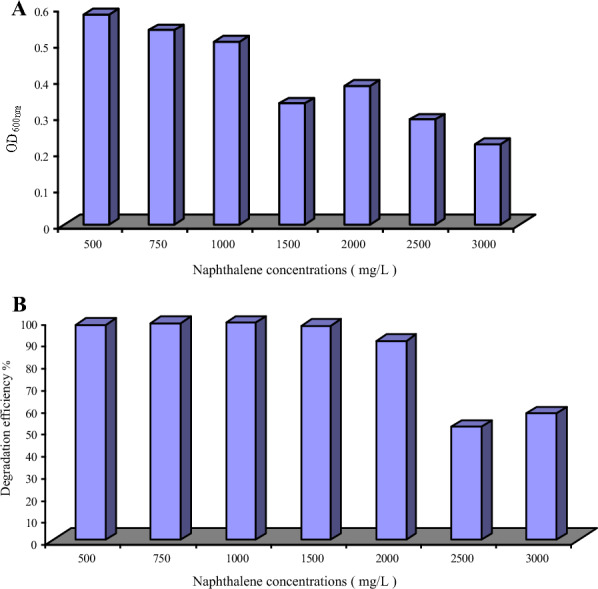
**A** Growth of *C. tropicalis* on different concentrations of naphthalene, **B** The degradation efficiency (%) of *C. tropicalis* on different concentrations of naphthalene

The degradation potency of the experimental strain (B) on naphthalene as a sole carbon source in sterile liquid sea water was studied. The NSW medium was supplemented with different naphthalene concentrations that ranged from 100 to 3000 mg/L. Each trial was inoculated with 2% preculture of the strain and incubated at 30 °C with a shaking speed of 200 rpm for 3 days. The growth was monitored by measuring the OD, while the residual naphthalene concentration was measured by GC. The observed strain B growth and naphthalene degradation efficiency (%) are presented in Fig. 4A, B, respectively. These results pointed out that, the ability of the strain to grow decreased with increasing naphthalene concentration. As shown in Fig. 4B, almost complete removal of naphthalene (98%) was recorded when the hydrocarbon concentration ranged between 500 to 1500 mg/L. Naphthalene concentrations higher than 2000 mg/L markedly reduced the removal efficiency (approximately 57%).

### Degradation of different phenol concentrations by *C. tropicalis* strain B

**Fig. 5:**
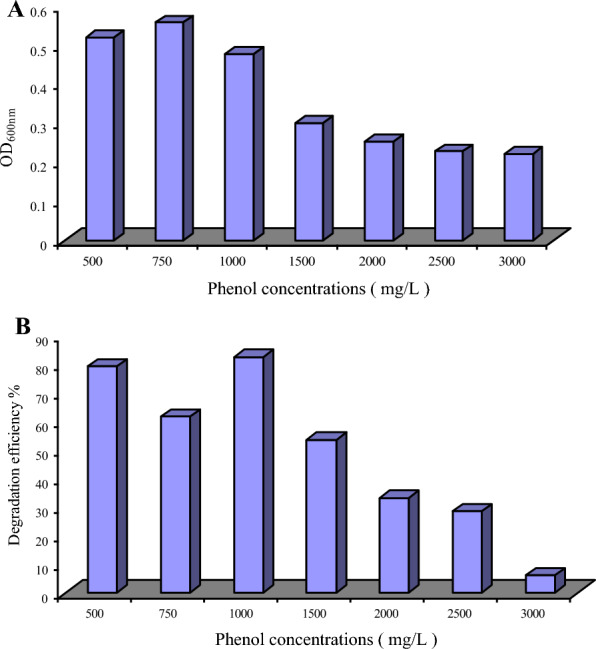
**A** Growth of *C. tropicalis* on different concentrations of naphthalene, **B** The degradation efficiency (%) of *C. tropicalis* on different concentrations of naphthalene

Phenol concentrations that ranged between 500 to 3000 mg /L were examined for degradation by *C. tropicalis*. The phenol was used as a sole carbon source in sterile sea water media, and inoculated with 2% preculture of tested strain and incubated at 30 °C with a shaking speed of 200 rpm for 3 days. The growth was monitored by measuring the OD, and the residual phenol was measured by GC. As shown in Fig. 5A, the ability of the strain to grow was markedly reduced with increasing phenol level especially at concentrations more than 1000 mg/L. The obtained results revealed also that maximum phenol removal (79.5%) was recorded by the yeast at concentrations between 500 to 1000 mg/L (Fig. 5B). The percentage of phenol removal was clearly decreased by the presence of higher phenol concentrations.

### Optimization of oil degradation by multi-factorial experiments

A sequential optimization approach was applied in this present study. The first phase deals with the screening for culture as well as nutritional factors affecting growth of strain with respect to increasing in oil degradation. The second approach is to optimize the factors that effectively control the biodegradation process.

### Evaluation of the factors affecting oil degradation using Plackett–Burman desing

**Fig. 6:**
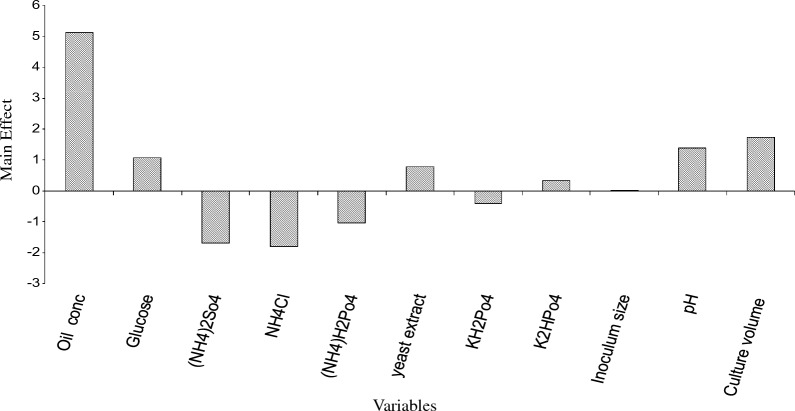
Effect of different culture factors on petroleum oil removal by C. *tropicalis* strain B in according to the results of the Plackett–Burman experiment

In the first approach, the Placket-Burman design was applied to reflect the relative importance of different factors. Eleven different factors (variables) including medium components were examined as independent variables. Table 1 shows the chosen factors and their levels. All experimental trials were performed in duplicates. The averages of the results of different trials (response) are given in grams and shown in Table 2. The main effect of each variable upon oil consumption was estimated as the differences between both averages of measurements made at the high level (+ 1) and at the low level (− 1) of the factor. The data in Table 2 show a wide variation from 1.02 g to 19.18 g of oil degradation. This variation reflects the importance of medium optimization to attain higher productivity. The analysis of the data from PBD involved a first order (main effects) model. The main effects of the examined factors on the oil degradation were calculated and presented graphically in Fig. 6**.** On the analysis of the regression coefficients of the eleven variables: Oil concentration, Glucose, yeast extract, K_2_HPo_4_, Inoculum size, pH, and culture volume showed positive effect on oil degradation. (NH_4_)_2_SO_4_, NH_4_Cl, (NH_4_)H_2_PO_4_, and KH_2_PO_4_ were contributed negatively.

**Table 1:** Independent variables and their levels test in the Plackett–Burman experiment

Independent variable	Component code	Low level (− 1)	High level (+ 1)
Oil concentration	X_1_	0.5%	1.0%
Glucose	X_2_	0.5%	1.0%
(NH_4_)_2_So_4_	X_3_	0.5%	1.0%
NH_4_Cl	X_4_	0.5%	1.0%
(NH_4_)H_2_Po_4_	X_5_	0.5%	1.0%
yeast extract	X_6_	0.1%	0.5%
KH_2_Po_4_	X_7_	0.1%	0.5%
K_2_HPo_4_	X_8_	0.1%	0.5%
Inoculum size	X_9_	1.0%	3.0%
pH	X_10_	5.0	7.0
Culture volume	X_11_	30 ml	50 ml

**Table 2:** The Plackett–Burman experimental design applied for evaluating factors influencing oil biodegradation by *C. tropicalis* strain B

Trials	Oil	Glucose	(NH_4_)_2_So_4_	NH_4_Cl	(NH_4_)H_2_Po_4_	yeast extract	KH_2_Po_4_	K_2_HPo_4_	Inoculum size	pH	Culture volume	Response B	Oil consumption (%)
1	+ 1	− 1	− 1	+ 1	− 1	+ 1	+ 1	+ 1	− 1	− 1	− 1	0.023	9.8
2	+ 1	− 1	+ 1	+ 1	+ 1	− 1	− 1	− 1	+ 1	− 1	− 1	0.007	2.98
3	+ 1	− 1	− 1	− 1	+ 1	− 1	− 1	+ 1	− 1	+ 1	+ 1	0.066	16.87
4	+ 1	+ 1	+ 1	− 1	− 1	− 1	+ 1	− 1	− 1	+ 1	− 1	0.03	12.78
5	− 1	− 1	+ 1	− 1	− 1	+ 1	− 1	+ 1	+ 1	+ 1	− 1	0.004	3.41
6	− 1	+ 1	− 1	+ 1	+ 1	+ 1	− 1	− 1	− 1	+ 1	− 1	0.003	2.56
7	+ 1	+ 1	− 1	− 1	− 1	+ 1	− 1	− 1	+ 1	− 1	+ 1	0.076	19.18
8	− 1	− 1	+ 1	− 1	+ 1	+ 1	+ 1	− 1	− 1	− 1	+ 1	0.001	0.51
9	+ 1	+ 1	+ 1	+ 1	+ 1	+ 1	+ 1	+ 1	+ 1	+ 1	+ 1	0.05	12.78
10	− 1	+ 1	− 1	− 1	+ 1	− 1	+ 1	+ 1	+ 1	− 1	− 1	0.002	1.7
11	− 1	− 1	− 1	+ 1	− 1	− 1	+ 1	− 1	+ 1	+ 1	+ 1	0.007	3.58
12	− 1	+ 1	+ 1	+ 1	− 1	− 1	− 1	+ 1	− 1	− 1	+ 1	0.002	1.02

### Optimization of the culture conditions by Box-Behnken design

**Fig. 7:**
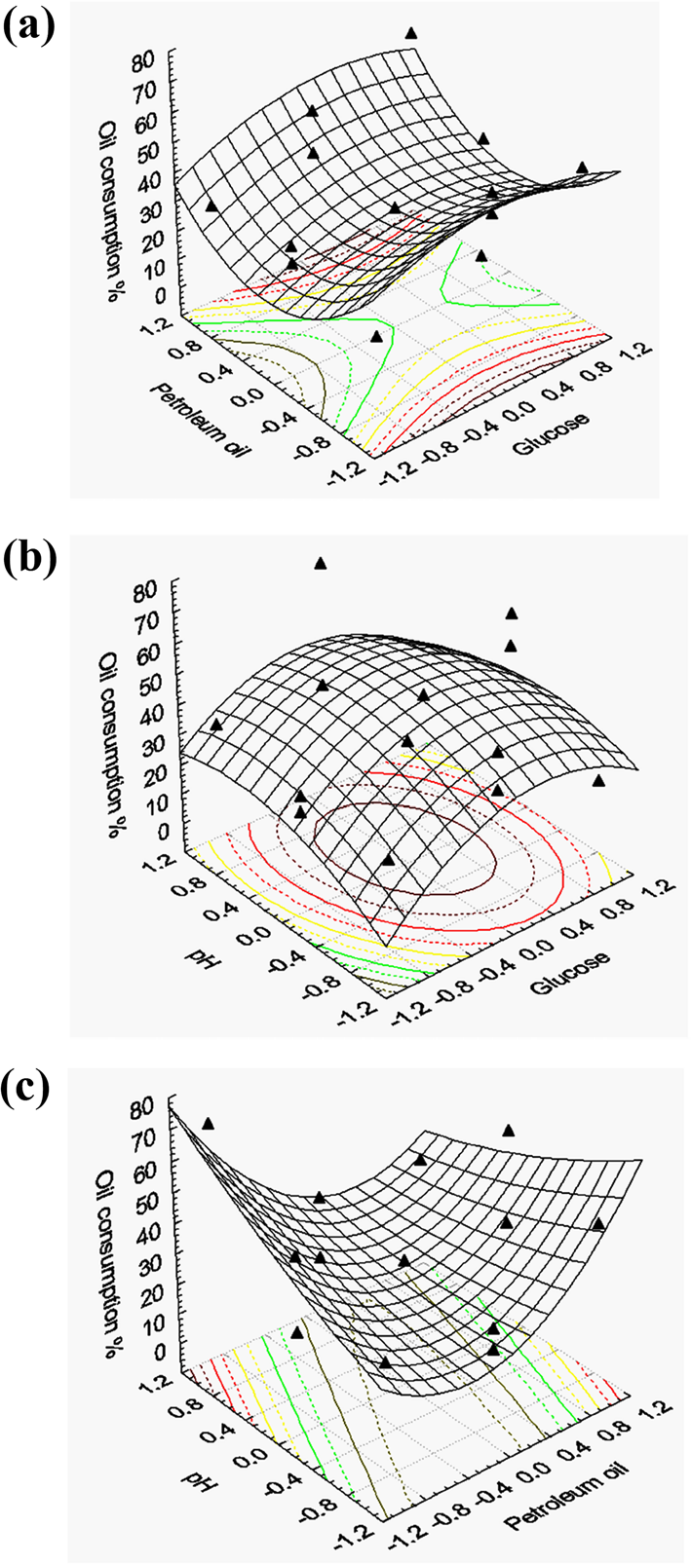
The response surface of oil consumption by *C. tropicalis* strain B as a function of **a** D-Glucose and oil, **b** oil and pH, **c** D-Glucose and pH in the culture environment. Triangular symbols represent the actual measured response data points

A second multi-factorial experiment was used according to the Box-Behnken design (a response surface methodology) to reach the optimum level of each of the effective independent variables to reach the maximum consumption or utilization of oil as response. Thirteen trial design matrix was applied to obtain a quadratic model for prediction of the optimum consumption of oil. In order to approach the the oil degradation optimum response region of the significant independent variables (D-Glucose X_1_; Oil X_2_; pH X_3_) were further explored, each at three levels. Table 3 represents the design matrix of the variables in together with the experimental results of the oil degradation. experimental results in the form of surface pilots were introduced (Fig. 7a) showed the higher levels of the oil degradation were attained with increasing the concentration of Oil, Glucose, and pH in the medium, (Fig. 7b and c). For predicting the optimal point, with Experimental constraints, a second-order polynomial function was fitted to the experimental results (linear optimization algorithm) of oil degradation:

$$ {\text{Y}}_{{\text{B}}} = \beta_{0} + \beta_{{1}} {\text{X}}_{{1}} + \beta_{{2}} {\text{X}}_{{2}} + \beta_{{3}} {\text{X}}_{{3}} + \beta_{{{12}}} {\text{X}}_{{1}} {\text{X}}_{{2}} + \beta_{{{13}}} {\text{X}}_{{1}} {\text{X}}_{{3}} + \beta_{{{23}}} {\text{X}}_{{2}} {\text{X}}_{{3}} + \beta_{{{11}}} {\text{X1}}^{{2}} + \beta_{{{22}}} {\text{X}}_{{2}}^{{2}} + \, \beta_{{{33}}} {\text{X}}_{{3}}^{{2}} $$

$$ {\text{Y}}_{{{\text{degradation}}}} { = 64}.{79} + {1}.0{\text{5125X}}_{{1}} + {2}.{\text{436X}}_{{2}} + {1}.{1}0{\text{25X}}_{{3}} + {2}.{\text{15X}}_{{1}} {\text{X}}_{{2}} - {7}.{\text{5475X}}_{{1}} {\text{X}}_{{3}} - 0.{\text{4575X}}_{{2}} {\text{X}}_{{3}} - {1}.{\text{625X}}_{{1}}^{{2}} - {6}.{\text{415X}}_{{2}}^{{2}} - 0.{\text{5275X}}_{{3}}^{{2}} $$

where, Y is the predicted response, β_0_ model constant; X_1_, X_2_, and X_3_ are the levels of independent variables; β_1_,β_2_,and β_3_ are linear coefficients; β_12_, β_13_, and β_23_, are cross product coefficients and β_11_,β_22_,and β_33_ are the quadratic coefficients. Microsoft Excel 97 was used for the regression analysis of the experimental data obtained. The quality of fitting of the polynomial model equation was expressed by the coefficient of determination R^2^. Where, X_1_, X_2_, and X_3_ are the Glucose, Petroleum oil, and pH respectively.

**Table 3:** Box-Behnken factorial experimental design, representing the response of oil degradation as influenced by glucose, petroleum oil_,_ and pH for *C. tropicalis* strain B

Trials	Variables			Oil consumption (%)
	X1	X2	X3	
1	+ 1	+ 1	0	54.9
2	+ 1	− 1	0	41.8
3	− 1	+ 1	0	18.8
4	− 1	− 1	0	12.5
5	+ 1	0	+ 1	0.7
6	+ 1	0	− 1	9.9
7	− 1	0	+ 1	25.0
8	− 1	0	− 1	18.0
9	0	+ 1	+ 1	22.6
10	0	+ 1	− 1	40.0
11	0	− 1	+ 1	71.0
12	0	− 1	− 1	25.0
13	0	0	0	22.0

Variables	Code		
	− 1	0	+ 1
Glucose (g%) (X1)	1	2	3
Petroleum oil (g%) (X2)	1	2	3
pH (X3)	6	6.5	7

Triple tests were performed and the average of three reading was considered

### Degradation of crude oil by immobilized yeast cells

**Fig. 8:**
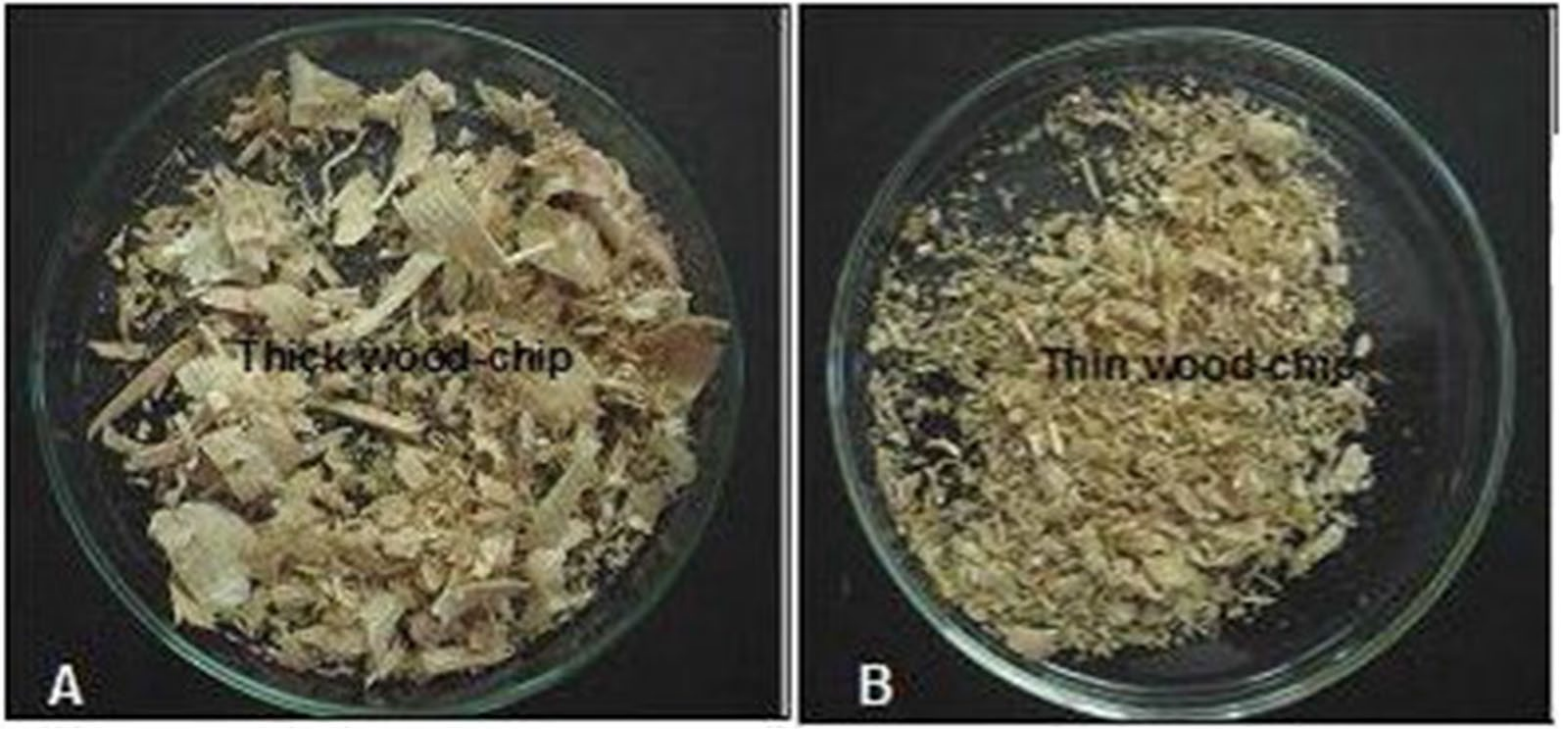
Thick wood chips (**A**) and thin wood chips (**B**)

**Fig. 9:**
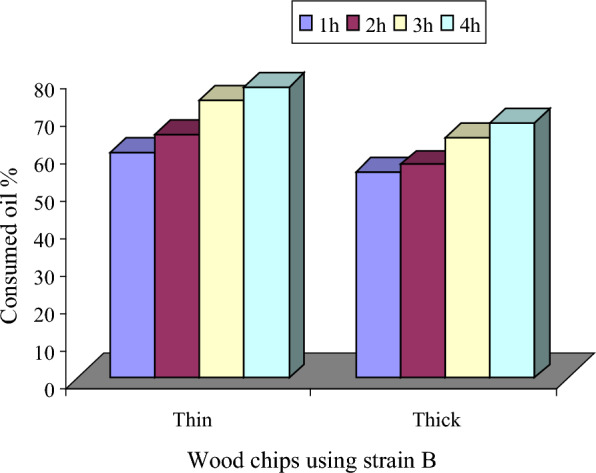
Oil consumption by yeast cells immobilized on thick and thin wood chips at different incubation times

The efficiency of oil degradation by immobilized yeast cells under tested conditions was measured and presented in Figs. 8, 9**.** The results showed that the use of thin wood chips with the yeast increased the removal percentage of oil than the thick wood chips. This figure shows also that, the more the incubation period, the more the amount of degraded oil. The maximum reached amount of oil removal (77.5%) was recorded by yeast cells immobilized on thin wood chips within 5 h.

### Degradation of crude oil by immobilized yeast using different weights of thin wood chips

This experiment was carried out to investigate the effect of using different weights of thin wood chips (0.5, 0.75, 1, 1.25, 1.5, 1.75 g/44cm^2^) on which cells were allowed to be immobilized for 3 h according to the preliminary experiments. Up to a weight of 1.25, the amount of oil removed from the aqueous medium in the absence of yeast cells was dependent upon the weight of the introduced wood chips (Table 4). It is worth to mention that 1.25 g of wood chips were enough to cover the base surface area of 250 ml Erlenmeyer conical flask. On the other hand, it had been observed that up to the highest examined level (1.75 g/ flask), the more the weight of wood chips carrying immobilized yeast cells, the more the amount of oil removal. This observation confirms the biological role of yeast cells in the process of oil degradation. However, the amounts of oil absorbed by wood chips were in general more than those consumed by yeast cells. Accordingly, further investigations for the pretreatment of wood chips are important to prevent, or at least reduce, the percentage of oil absorption by wood.

**Table 4:** Oil removal by different weights of wood chips and wood chips carrying immobilized yeast

Weight of chips (g)		Oil removal (%)		
		By wood chips and immobilized cells	By wood chips only	By immobilized cells only
0.50		33	19	14
0.75		68	36	32
1.00		69	36	30
1.25		74	52	22
1.50		76	53	23
1.75		89	53	36

### Microscopic examination of cells immobilized on thin wood chips

**Fig. 10:**
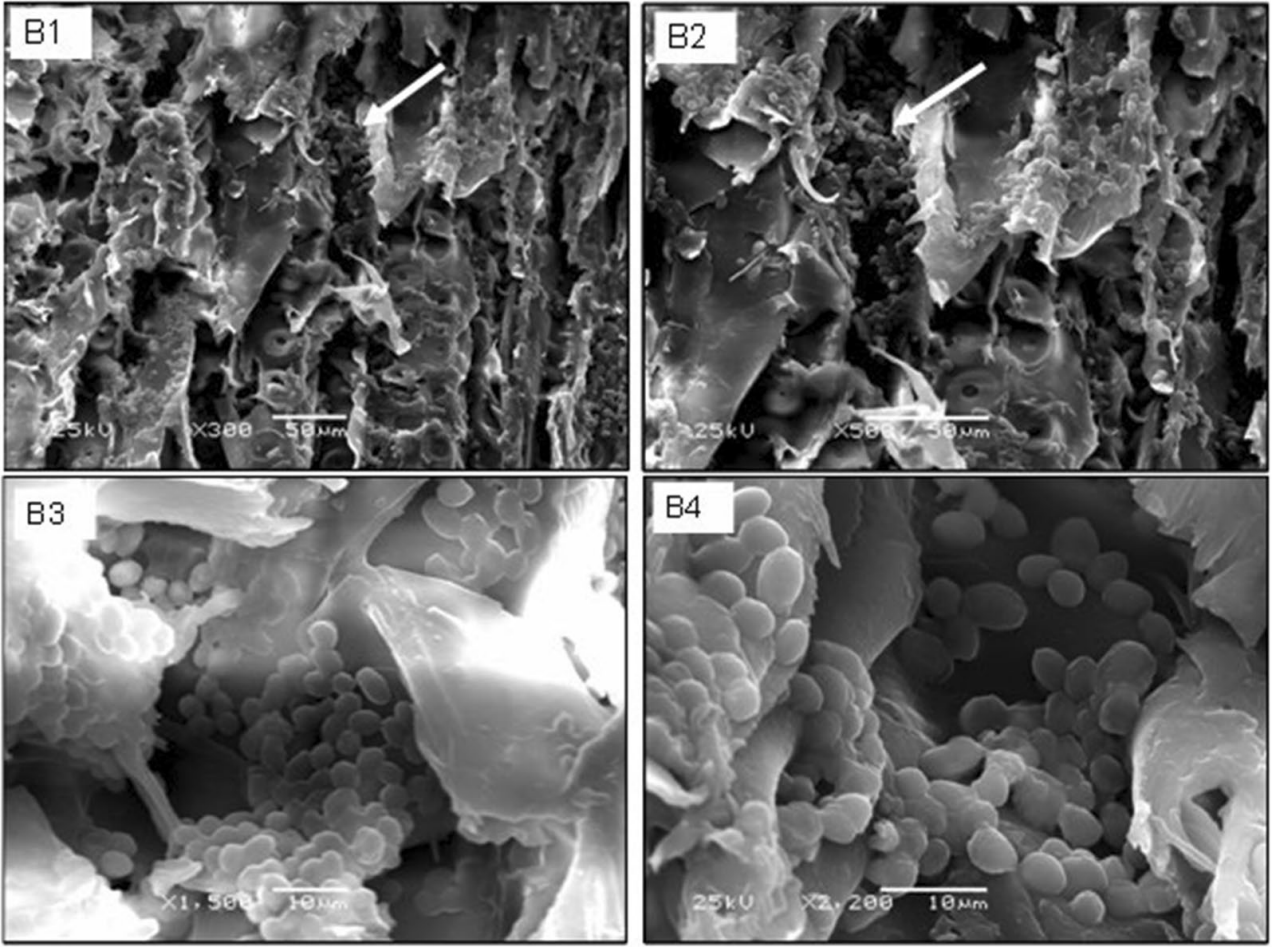
Scanning electron micrograph (SEM) showing adsorbed yeast on wood chips units. arrows indicate the focused unit. B1: X300; B2: X500; B3: X1500; B4: X2200

**Fig. 11:**
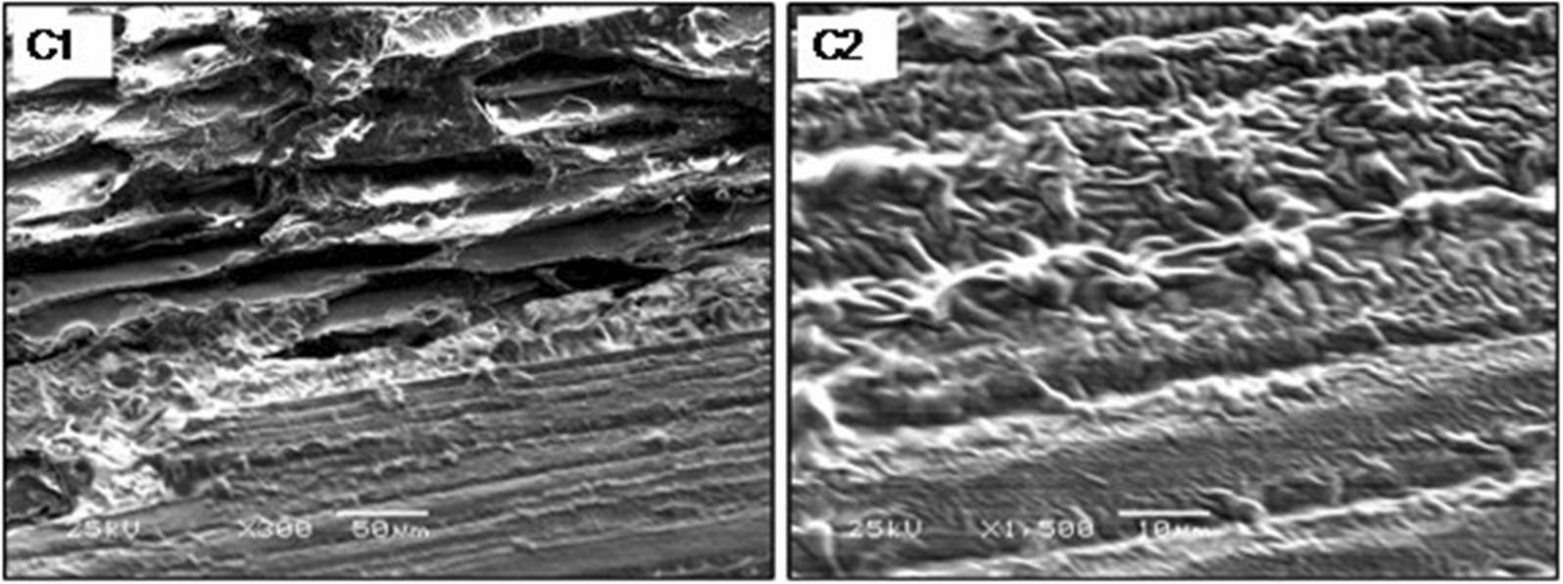
Photomicrograph (SEM) of wood chips surface with crude oil as a negative control. C1 X300; C2 X1500

The cells adsorbed on thin wood chips were examined by scanning electron microscopy at different magnification folds. Cell agglutination was easily recognized on the surface of the examined wood chip (Fig. 10). On the other hand, a negative control showed oil droplets attached to the solid surface of the assayed carrier (Fig. 11).

## Discussion

The rapid oil industry increase has a major drawback on the human and other living organisms’ life. Bioremediation process include the use of living microorganisms to transform these dangerous organic hydrocarbons into carbon dioxide [24–26]. These living microorganisms should be able to live and adapted to the environment that will be remediate. The present study aimed to isolate and purify local yeast strains from oil contaminated marine water samples that are able to aerobically degrade crude petroleum oils and its organic derivatives and utilize them as a sole carbon and energy sources. The yeast strain that fulfilled the goal (isolate B) was identified as a *Candida tropicalis* strain based on a 99% 18S rDNA similarity. Preliminary physiological characterization studies proved that the selected yeast strain has the ability to grow over a wide range of pH (4–11) with optimal growth at pH 4 and can tolerate salt concentrations in the range of 1–12%. In addition to being among the natural microbial flora of petroleum oil polluted environments, these properties, motivated us to study the potentiality of this strain to be used for biodegradation petroleum oil in sea water. Aeration plays an important role in oil bioremediation process and is considered as limiting factor in the biodegradation process of marine oil spills where, oxygen is the main factor necessary for the initial utilization and breakdown of organic hydrocarbon and the subsequent reactions [27]. Thus, all liquid cultures applied in this work were incubated in a rotary shaker at 200 rpm. The results section suggested that medium containing glucose and yeast extract supported growth as well as petroleum oil consumption percentage. On the other hand, a medium that lacks glucose or yeast extract but contains petroleum oil showed low levels of oil consumption percentage. This could be recognized to the carbon–nitrogen ratio importance in controlling fermentation reactions as well as the growth. Using growth media with enough glucose concentration and nitrogen deficiency, it is expected that most important enzymes required for degrading petroleum oil are not sufficiently expressed. Thus, the presence of glucose and yeast extract in the medium enhanced large biomass formation and consequently the biodegradation capacity of the degradable yeast cells. In addition to amino acids (the building units of enzymes), yeast extract contains vitamins and other substances that act as co-factors which could contribute to the production of many enzymes [28]. Scanning electron microscopy indicated that the yeast cellular diameter was changed according to medium composition. The complex medium (M6) supported cellular enlargement much more than the relatively poor medium (M2). This result confirms the importance of organic nitrogenous sources for enhancing initially the growth as previously described. The present study focused also on studying the ability of the experimental yeast strain to degrade different aliphatic and aromatic hydrocarbons and some their derivatives, each as a sole energy and carbon source in a natural sea water medium. Among tested compounds the tested yeast showed a great ability to degrade most of them, but with distinctive preference for naphthalene and phenol. Regarding the aliphatic hydrocarbons, the strain showed varied potential degradation which decreases with increasing the chain length (C6, C7 and C15). Also, Adeleye et al., investigated that the yeasts showed a high ability to degrade most of the tested organic compounds but showed a distinct preference for hexadecane. Besides their ability to grow on hexadecane, the three isolated yeasts showed suitable potential to degrade and grow on some different refinery subproducts, such as diesel fuel, crude oil, and Undecane. Measurement of the biomass of these isolates, using undecane, hexadecane, diesel oil, and crude oil as organic substrates indicated that the yeasts were able of degrading and utilizing a wide range of intermediate carbon chain length *n*-alkanes, probably because of the less toxic nature of the long-chain *n*-alkanes [29]. Two multi-factorial sequential experimental optimization approaches were applied in this work. These included the Plackett–Burman and Box-Behnken designs. The results indicated that oil concentration was the most effective independent variable with respect to oil bioremediation by *Candida tropicalis* strain B. The results also showed that the high levels of some variables such as oil and glucose concentrations, culture volume, pH, yeast extract and K_2_HPO_4_ were nearer to optimum. Meanwhile, the low levels of other factors such as ammonium sulfate and ammonium chloride were found to be closer to optimum. It is likely that the importance of glucose, an easily utilizable carbon source, which occurs at the initial stage of the culture to support biomass formation. However, in many cases the cells start to utilize oil only when simple sugars became depleted [30–32]. It has been previously reported that the lack of nitrogen and phosphorus limit biodegradation [33]. Yeast extract (a good source of amino acids, vitamins and co-factors) supports growth and allows the production of enzymes and other proteins [28]. The presence of phosphate plays a critical role where inadequate supply of this nutrient may result in slowing down of all metabolic reactions. In addition to its importance in the formation of ATP (the main energy carrier biomolecule), phosphate plays a critical role in signal transduction which mediates environmental adaptation of the microbial cells [34]. The capacity of immobilized bacteria and yeast [35]. to degrade efficiently petroleum hydrocarbons have been reported. In this work, degradation of crude oil by yeast cells immobilized on thin and thick wood chips for different incubation times. The results showed that thin wood chips are better. Most likely, this observation is a result of the increased surface area of the thin wood chips which allowed attachment of more yeast cells.

## Conclusion

This study focused on the isolation and characterization of *Candida tropicalis* for the biodegradation of petroleum oil in marine environments. The research highlighted the importance of environmental protection laws in regulating waste organic materials dumping and the global dependence on crude petroleum oil. Bioremediation, using living microorganisms, was identified as a promising approach to mitigate the harmful effects of petroleum hydrocarbons. The isolated yeast strain*, Candida tropicalis* (isolate B), demonstrated favorable characteristics for petroleum oil biodegradation. Physiological characterization revealed its ability to thrive in a wide pH range and tolerate varying salt concentrations, making it well-adapted to marine environments. The presence of glucose and yeast extract in the growth medium significantly enhanced the strain's biomass formation and biodegradation capacity. The yeast strain exhibited remarkable capabilities in degrading various aliphatic and aromatic hydrocarbons, with a notable preference for naphthalene and phenol. Optimization experiments highlighted the influential role of oil concentration on the bioremediation efficiency of *Candida tropicalis* strain B. The research also explored the use of immobilized yeast cells on thin wood chips, which demonstrated enhanced crude oil degradation compared to thick wood chips, likely due to increased surface area for cell attachment. Overall, the findings of this study contribute to the understanding of *Candida tropicalis'* potential for petroleum oil bioremediation in marine environments. The research provides insights into the physiological characteristics, substrate preferences, and optimization parameters for the efficient degradation of petroleum hydrocarbons. These findings have implications for developing sustainable approaches to address oil pollution and promote environmental conservation in oil-contaminated marine ecosystems. Further research and application of this yeast strain in real-world scenarios could lead to effective and eco-friendly strategies for mitigating the impacts of petroleum oil spills.

## Data Availability

All the data generated during this study are included in this published article.
