# Correction: Isolation and characterization of Candida tropicalis B: a promising yeast strain for biodegradation of petroleum oil in marine environments

**DOI:** 10.1186/s12934-024-02348-7

**Published:** 2024-03-28

**Authors:** Ghada E. Hegazy, Nadia A. Soliman, Soha Farag, Ehab R. El-Helow, Hoda Y. Hassan, Yasser R. Abdel-Fattah

**Affiliations:** 1 National Institute of Oceanography & Fisheries, NIOF-Egypt, Qaitbay Sq, El-Anfoushy, 11865 Alexandria, Egypt; 2 Bioprocess Development Department, Genetic Engineering & Biotechnology Research Institute (GEBRI), City of Scientific Research & Technological Applications (SRTA-City), New Borg Elarab City, Alexandria Egypt; 3 Environmental Biotechnology Department, Genetic Engineering & Biotechnology Research Institute (GEBRI), City of Scientific Research &Technological Applications (SRTA-City), New Borg Elarab City, Alexandria Egypt; 4 Botany & Microbiology Department, Faculty of Science, Alexandria University, Alexandria, Egypt

**Correction: Microbial Cell Factories (2024) 23:20**

10.1186/s12934-023-02292-y

In this article, the author name Hoda H. Yusef was incorrectly written as Hoda Y. Hassan. The last statement in the author contributions was incorrectly given as “YRA suggested the main point of his work” but should have been “YRA suggested the main point of this work”. The original article has been corrected.

